# Supplementary material for: Spatial distribution and factors associated with co-occurrence of anemia and undernutrition among children aged 6–59 months in East Africa
Source: PLoS One. 2026 Feb 13;21(2):e0342437. doi: 10.1371/journal.pone.0342437 (PMC12904427; doi:10.1371/journal.pone.0342437)
Supplement: S1 File — This file contains one table and multiple figures related to the spatial distribution and analysis of the co-occurrence of anemia and undernutrition among children aged 6–59 months in East Africa.S1 Fig A. Spatial distribution of co-occurrence of anemia and undernutrition. S1 Fig B. Incremental autocorrelation for the co-occurrence of anemia and undernutrition. S1 Fig C. Ordinary Kriging interpolation for the co-occurrence of anemia and undernutrition. S1 Table A. Spatial SaTscan analysis results for the co-occurrence of anemia and undernutrition. (DOCX) [file pone.0342437.s001.docx]

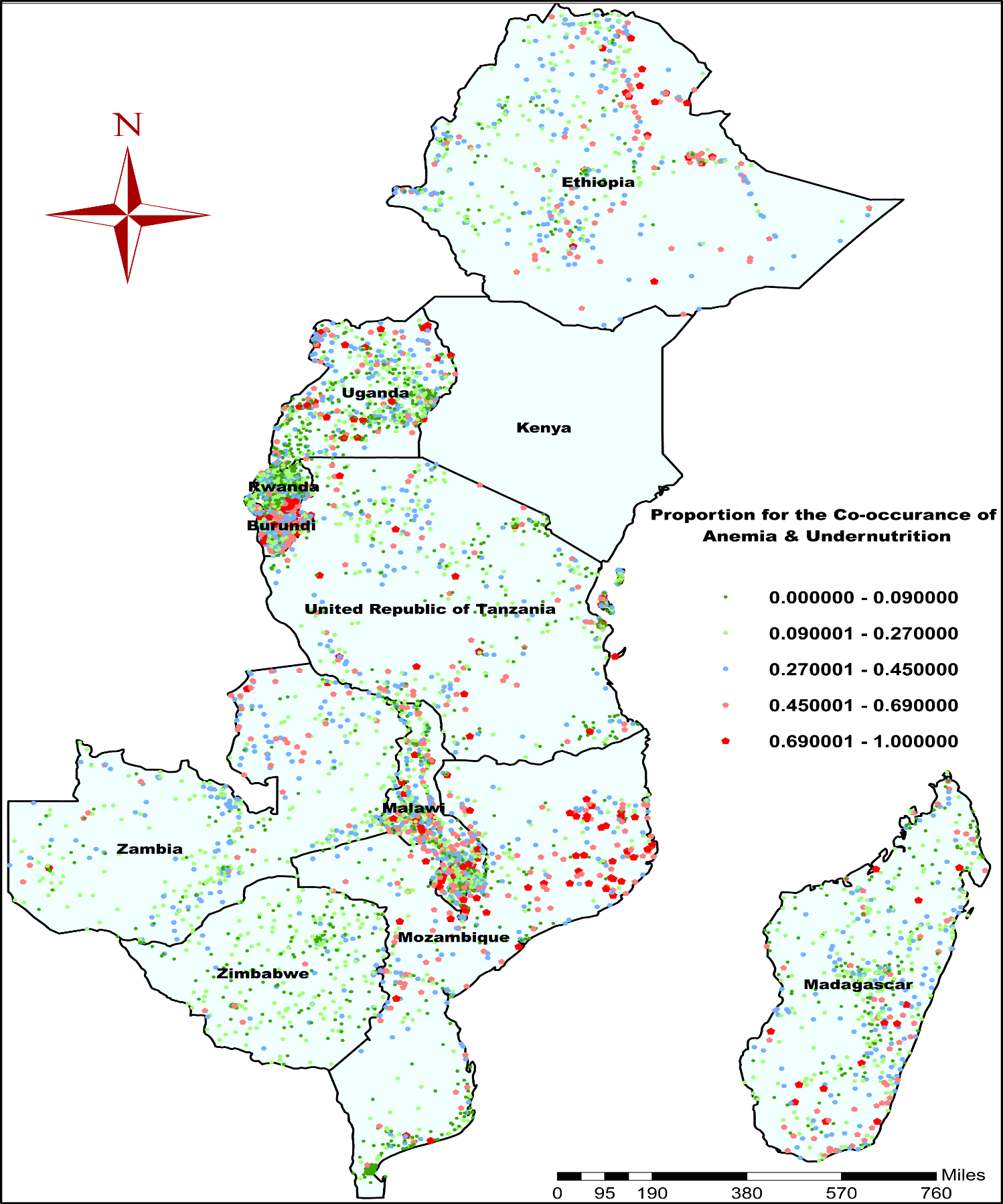


Fig A. Spatial distribution of co-occurrence of anemia and undernutrition among children 6-59 months of age in East Africa. **Source:** Administrative boundary shapefile obtained from OpenAfrica (<https://open.africa/dataset/africa-shapefiles>). [**Note**: The authors utilized the shapefile solely as a basemap, performing all spatial processing, analysis, visualization, and modifications for illustrative and analytical purposes only].


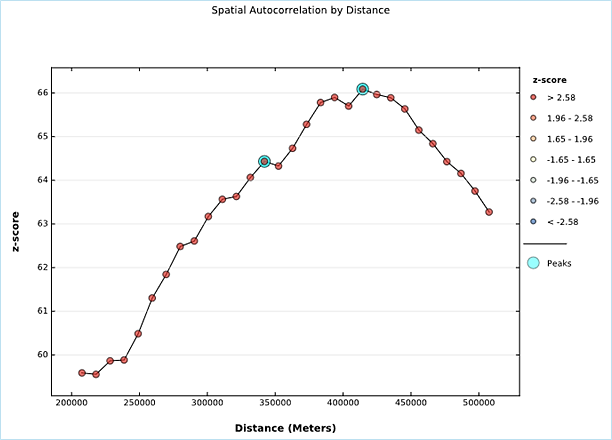


Fig B. Incremental Autocorrelation of co-occurrence of anemia and undernutrition among children 6-59 months in East Africa.


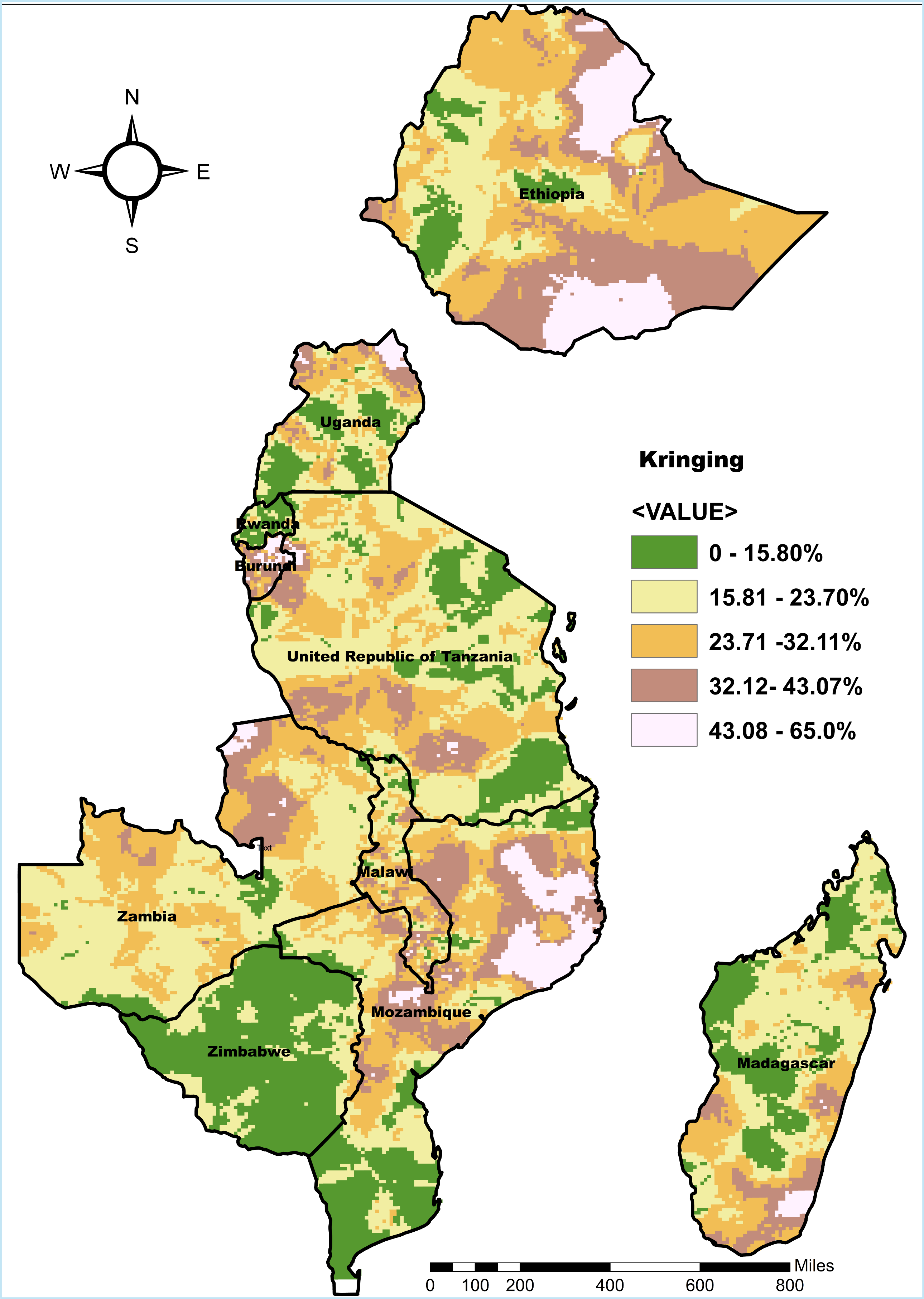


Fig C. Ordinary Kriging interpolation of co-occurrence of anemia and undernutrition among children aged 6-59 months in East Africa. **Source:** Administrative boundary shapefile obtained from OpenAfrica (<https://open.africa/dataset/africa-shapefiles>). [**Note**: The authors utilized the shapefile solely as a basemap, performing all spatial processing, analysis, visualization, and modifications for illustrative and analytical purposes only].

Table A. Spatial SaTscan analysis result of co-occurrence of anemia and undernutrition among children aged 6-59 months in East Africa.

| Cluster Windows | Total Location IDs | Coordinates / Radius | RR | LLR | P-Value |
| --- | --- | --- | --- | --- | --- |
| 1 | 146 | (3.22°S, 30.54°E) / 97.42 km | 3.48 | 82.53 | <0.001 |
| 2 | 100 | (15.25°S, 39.35°E)/271.52 km | 3.37 | 42.76 | <0.001 |
| 3 | 118 | (18.36°S, 30.27°E)/274.04 km | 0.045 | 38.69 | 0.001 |
| 4 | 97 | (11.38°N, 41.70°E)/229.44 km | 3.29 | 26.03 | 0.001 |
| 5 | 159 | (1.90°S, 30.04°E) / 64.56 km | 0.16 | 25.83 | 0.001 |
| 6 | 178 | (13.45°S, 25.84°E)/447.41 km | 0.18 | 23.80 | 0.001 |
| 7 | 151 | (1.64°N, 32.71°E) / 169.65 km | 0.36 | 12.59 | 0.027 |
