# Supplementary material for: Spatial distribution and factors associated with co-occurrence of anemia and undernutrition among children aged 6–59 months in East Africa
Source: PLoS One. 2026 Feb 13;21(2):e0342437. doi: 10.1371/journal.pone.0342437 (PMC12904427; doi:10.1371/journal.pone.0342437)
Supplement: S2 File — This file contains additional outputs from the spatial regression analyses for the co-occurrence of anemia and undernutrition among children aged 6–59 months in East Africa. S2 Table A: Summary statistics of the estimated coefficients for local terms in GWR model. S2 Fig A: MGWR coefficient estimates for children perceived as having smaller/very small birth size for the co-occurrence of anemia and undernutrition. S2 Fig B: MGWR coefficient estimates for maternal age 15–24 years for the co-occurrence of anemia and undernutrition. S2 Fig C: MGWR coefficient estimates of mothers with no formal education for the co-occurrence of anemia and undernutrition. S2 Fig D: MGWR coefficient estimates of multiple birth for the co-occurrence of anemia and undernutrition. (DOCX) [file pone.0342437.s002.docx]

**Supplementary file 2**

Table A. The summary statistics of the estimated coefficients of the local terms (GWR model).

| **Variable** | **Mean** | **STD** | **Min** | **Median** | **Max** | **Bandwidth** |
| --- | --- | --- | --- | --- | --- | --- |
| Intercept | -0.022 | 0.162 | -0.362 | 0.005 | 0.377 | 716.0 |
| Proportion of maternal anemia | 0.158 | 0.085 | -0.006 | 0.157 | 0.372 | 716.0 |
| Proportion of no Vitamin A supplementation | 0.059 | 0.054 | -0.158 | 0.064 | 0.244 | 716.0 |
| Proportion of having Diarrhea | 0.020 | 0.056 | -0.143 | 0.016 | 0.220 | 716.0 |
| Proportion of having a Fever | 0.093 | 0.067 | -0.040 | 0.085 | 0.342 | 716.0 |
| Proportion of tobacco user/smoker mothers | -0.016 | 0.117 | -0.449 | 0.019 | 0.401 | 716.0 |
| Proportion of the poorest household | 0.072 | 0.086 | -0.196 | 0.095 | 0.262 | 716.0 |
| Proportion of the poorer household | 0.059 | 0.062 | -0.101 | 0.057 | 0.269 | 716.0 |
| Proportion of no media exposure | 0.029 | 0.073 | -0.205 | 0.040 | 0.260 | 716.0 |
| Proportion of birth interval <24 months, | 0.046 | 0.054 | -0.091 | 0.054 | 0.162 | 716.0 |
| Proportion of maternal age 15-24 | 0.038 | 0.059 | -0.140 | 0.027 | 0.201 | 716.0 |
| Proportion of no maternal education | 0.153 | 0.094 | -0.193 | 0.166 | 0.385 | 716.0 |
| Proportion of multiple births | 0.055 | 0.051 | -0.123 | 0.052 | 0.216 | 716.0 |
| Proportion of overweight mothers | -0.082 | 0.062 | -0.341 | -0.067 | 0.029 | 716.0 |
| Proportion of no health insurance | 0.084 | 0.071 | -0.178 | 0.087 | 0.423 | 716.0 |
| Proportion of female sex | -0.053 | 0.043 | -0.166 | -0.059 | 0.091 | 716.0 |
| Proportion of children aged 12-23 months | 0.062 | 0.041 | -0.058 | 0.066 | 0.203 | 716.0 |
| Proportion of smaller/very small perceived birth size | 0.041 | 0.054 | -0.137 | 0.046 | 0.206 | 716.0 |


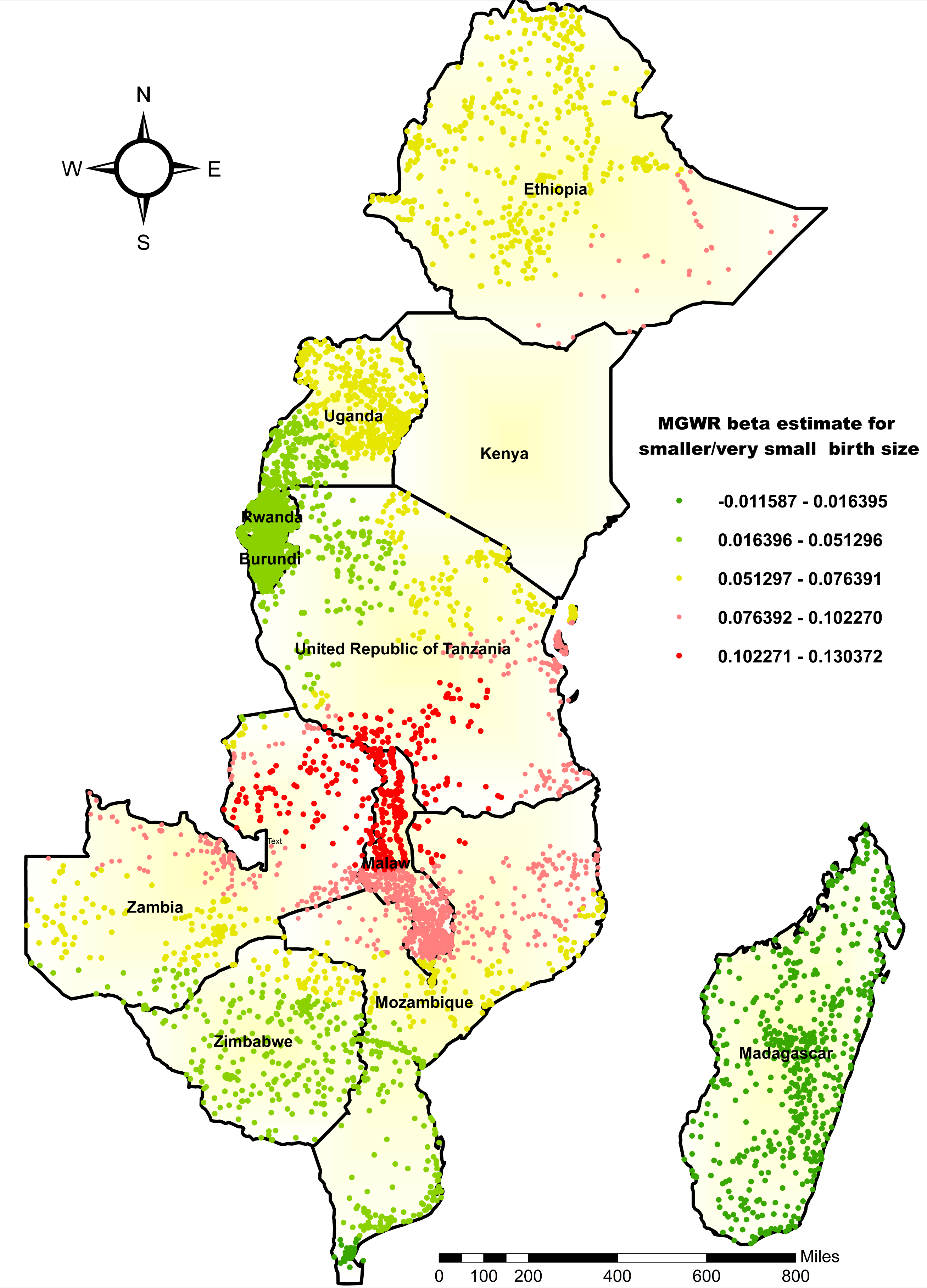


Fig A. MGWR coefficient estimates of being perceived as having a smaller/very small birth size for the co-occurrence of anemia and undernutrition among children 6-59 months in East Africa. **Source**: Administrative boundary shapefile obtained from OpenAfrica (https://open.africa/dataset/africa-shapefiles). [**Note**: The authors utilized the shapefile solely as a basemap, performing all spatial processing, analysis, visualization, and modifications for illustrative and analytical purposes only].


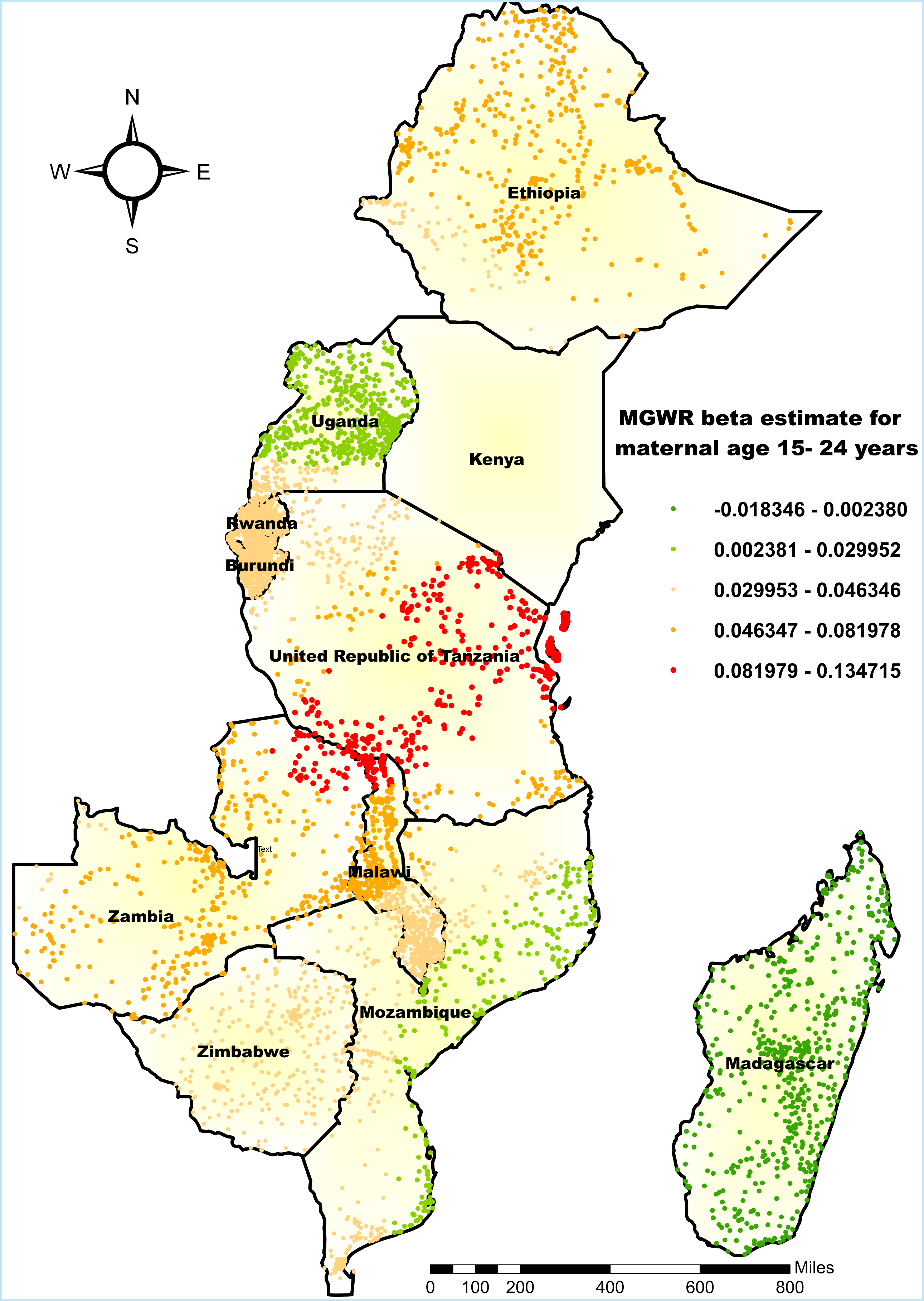


Fig B. MGWR coefficient estimates of maternal age 15-24 years for co-occurrence of anemia and undernutrition among children 6-59 months in East Africa. **Source**: Administrative boundary shapefile obtained from OpenAfrica (https://open.africa/dataset/africa-shapefiles). [**Note**: The authors utilized the shapefile solely as a basemap, performing all spatial processing, analysis, visualization, and modifications for illustrative and analytical purposes only].


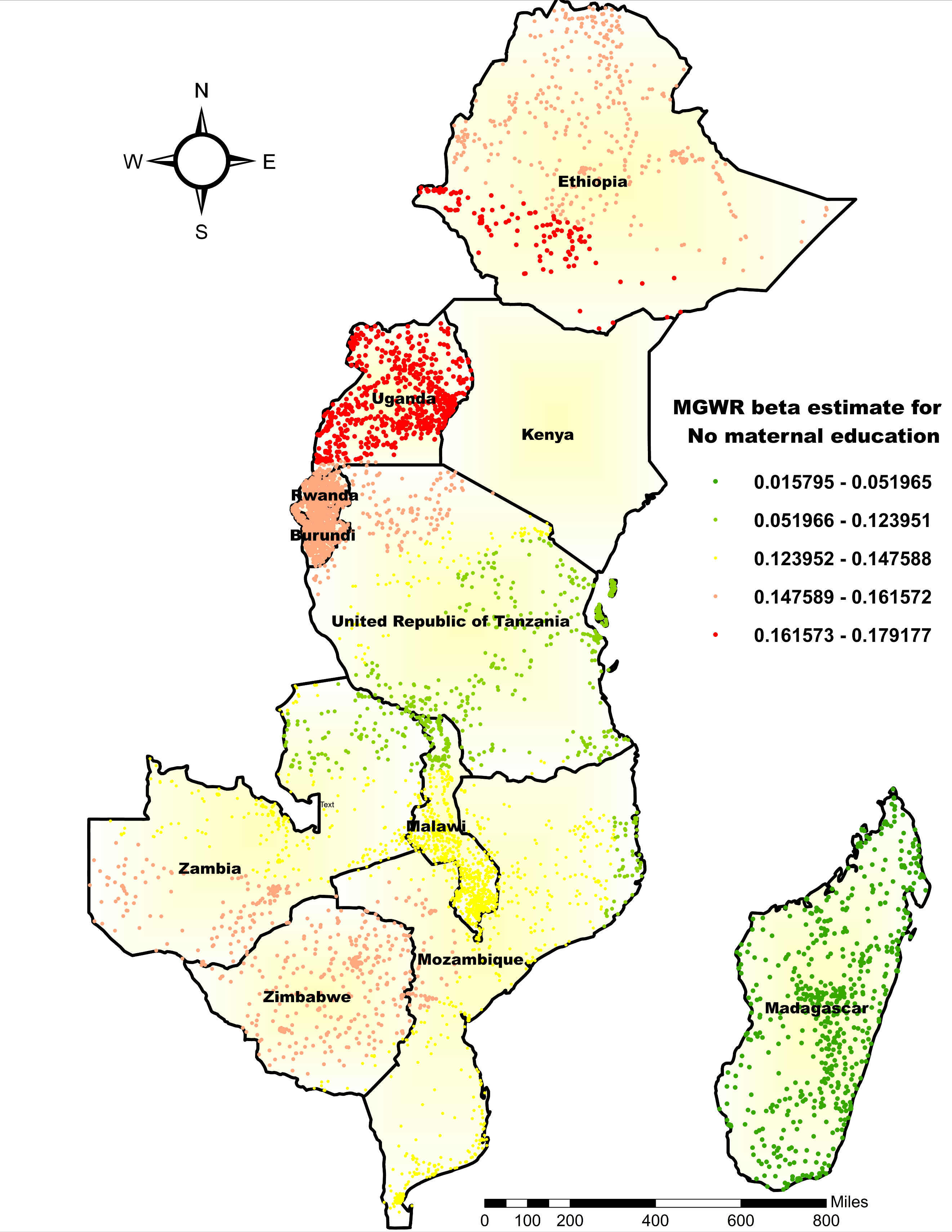


Fig C. MGWR coefficient estimates no maternal education for the co-occurrence of anemia and undernutrition among children 6-59 months in East Africa. **Source**: Administrative boundary shapefile obtained from OpenAfrica (https://open.africa/dataset/africa-shapefiles). [**Note**: The authors utilized the shapefile solely as a basemap, performing all spatial processing, analysis, visualization, and modifications for illustrative and analytical purposes only].


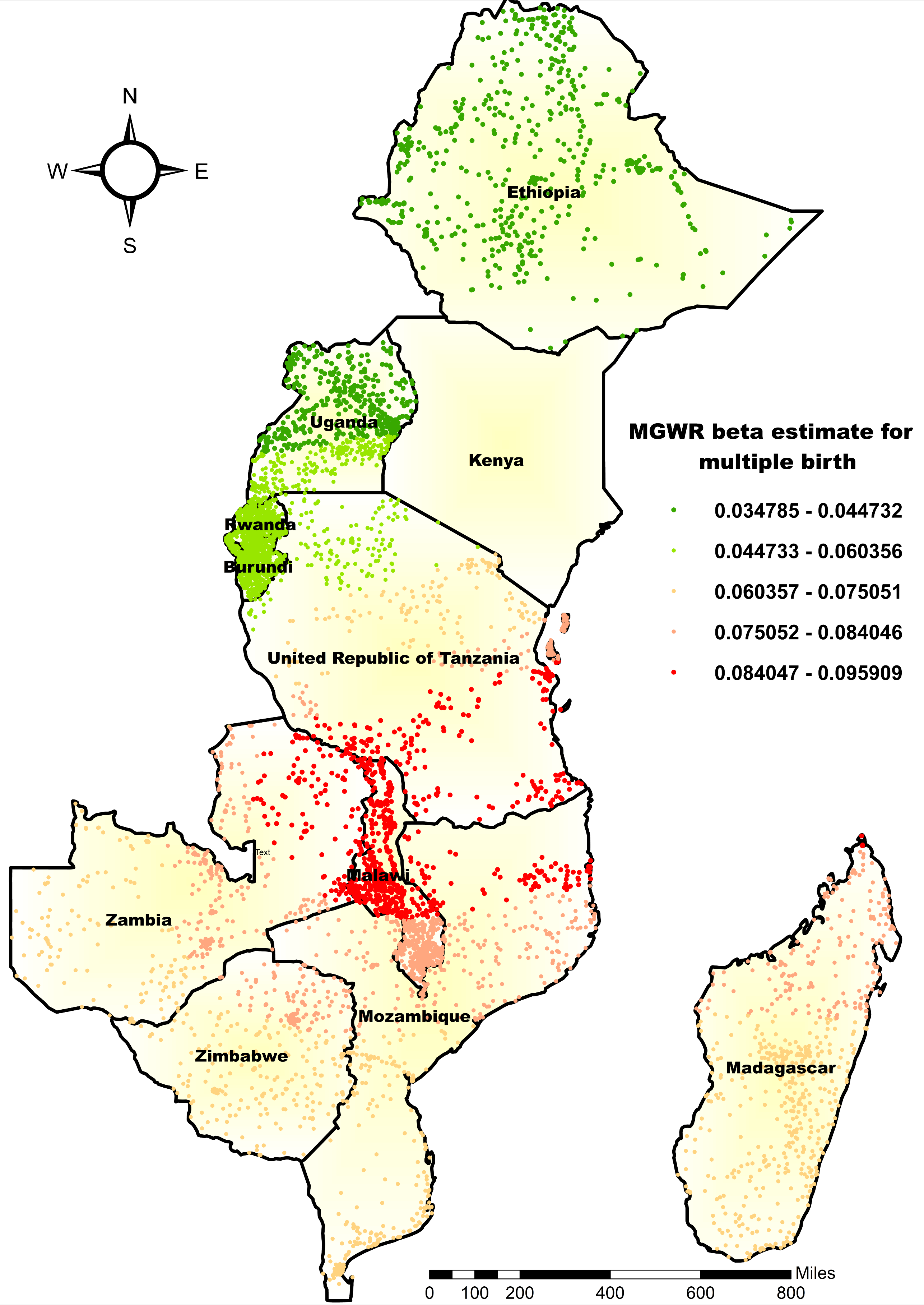


Fig D. MGWR coefficient estimates of multiple birth for the co-occurrence of anemia and undernutrition among children 6-59 months in East Africa. **Source**: Administrative boundary shapefile obtained from OpenAfrica (https://open.africa/dataset/africa-shapefiles). [**Note**: The authors utilized the shapefile solely as a basemap, performing all spatial processing, analysis, visualization, and modifications for illustrative and analytical purposes only].
