# Supplementary material for: Spatial distribution and factors associated with co-occurrence of anemia and undernutrition among children aged 6–59 months in East Africa
Source: PLoS One. 2026 Feb 13;21(2):e0342437. doi: 10.1371/journal.pone.0342437 (PMC12904427; doi:10.1371/journal.pone.0342437)
Supplement: Attachment — Submitted filename: Comments to the authors.docx [file pone.0342437.s004.docx]

Comments to the authors:

Line 346 table 2,

Please carefully recheck and revise the percentage calculations in the tables, as several variables do not sum to 100%, including maternal literacy, maternal anemia, drinking water, sanitary facilities, birth order, and cluster altitude.

You have specified the anemia cutoff for children under five; however, the cutoff used to define maternal anemia has not been clearly stated.

Line 501-505

The observed inverse association between the lack of vitamin A supplementation and the co-occurrence of anemia and undernutrition should be clearly explained and contextualized in the Discussion section, with reference to existing evidence.

Line 512-516

The inverse association between recent diarrhea and the co-occurrence of anemia and undernutrition is contrary to existing evidence. This finding should be critically explained and discussed in the Discussion section.

Line 679-684

While the explanation focusing on low maternal iron stores and reduced caregiving capacity is valid, the discussion should also consider broader contextual factors. Maternal anemia may reflect underlying household food insecurity and suboptimal maternal dietary practices, which can directly influence child feeding practices and contribute to the co-occurrence of anemia and undernutrition in children. These pathways should be acknowledged and discussed.

Line 793 conclusion

The recommended strategies in the Conclusion should be supported by relevant evidence or successful intervention examples in the Discussion.
